# Effects of physical activity, social capital on positive emotions in older adults—A study based on data from the 2022 CFPS survey

**DOI:** 10.3389/fpsyg.2025.1554741

**Published:** 2025-04-09

**Authors:** Long Niu, Xiaodong Zhang, Yichen Ma

**Affiliations:** ^1^Center for Physical Education, Xi'an Jiaotong University, Xi'an, China; ^2^Department of Physical Education, Guangzhou Xinhua University, Guangzhou, China; ^3^Department of Sociology, School of Humanities and Social Sciences, Xi'an Jiaotong University, Xi'an, China

**Keywords:** older adults, physical activity, individual social capital, collective social capital, positive emotions

## Abstract

**Background:**

Existing literature indicates that physical activity has a significant positive impact on the positive emotions in older adults; however, the mechanism of the relationship between physical activity and positive emotions remains unclear. This study aims to explore the role of social capital in the relationship between physical activity and positive emotions among older adults.

**Methods:**

First, the positive mood indicators of the elderly were constructed through the Euclidean distance method, and descriptive statistics and correlation analyses were performed on the relevant variables involved in this paper. Secondly, structural equation (SEM) was used to establish a parallel mediation model to analyze the mediating effect of social capital between physical activity and positive emotions of the elderly. Meanwhile, in order to test the potential influence of gender on the mediating path, this study used Multi-group SEM to divide the samples into two groups of males and females, and compared the path coefficients between groups through the Likelihood Ratio Test (LRT). Finally, the average treatment effect of ATT, i.e., the experimental group, was obtained through the propensity score matching test.

**Result:**

The results show that: (1) Physical activity has a significant positive effect on the positive emotions of older adults. (2) Social capital (i.e., individual social capital and collective social capital) significantly positively influences the positive emotions of older adults. (3) Physical activity has a significant positive effect only on collective social capital, and the mediation analysis reveals that collective social capital plays a significant mediating role in the relationship between physical activity and positive emotions in older adults.

**Conclusion:**

This study reveals that physical activity not only directly enhances the positive emotions of older adults, but also exerts an indirect effect by strengthening collective social capital. Therefore, it is recommended that the Chinese government promote the social connections and collective interactions of older adults by providing public spaces, organizing community activities, and strengthening social interaction platforms. These efforts could help improve the positive emotions of older adults and contribute to the achievement of healthy aging goals.

## 1 Introduction

With the intensification of global population aging, the mental health issues of older adults have become a significant public health concern. This is particularly true for China, which is undergoing a rapid and intense process of societal aging. According to the “2022 Statistical Bulletin on the Development of Civil Affairs” released by the Ministry of Civil Affairs, the population of individuals aged 60 and above in China reached 280 million in 2022, accounting for 19.8% of the total population. As the aging process accelerates, the mental health issues of older adults in China are becoming increasingly prominent, with mood disorders, particularly depression, emerging as one of the most common psychological issues among Chinese seniors (Zhang et al., 2020). It seriously affects the late life of the elderly. And in order to reduce negative emotions in individual emotional states, academics have focused on positive emotions in older adults, i.e., subjective feeling emotional states that include pleasure, happiness, and contentment (Vanderlind et al., 2022; Sturm et al., 2022). Relevant studies have shown that the cultivation and maintenance of positive emotions are important for enhancing the quality of life of older adults, promoting social participation, and reducing the incidence of mental illness (Silton et al., 2020). It not only helps older adults to better cope with life challenges, but also has a profound impact on their physical health and social adaptability (Fingerman et al., 2021; Shiota et al., 2021). However, how to effectively enhance positive emotions in older adults remains a major challenge in current academic research and policy practice.

Positive emotions are not only a psychological experience but also a social phenomenon that. On the one hand, they are significantly influenced by an individual's social network, interpersonal relationships, and social capital. On the other hand, the positive emotions of older adults are also constrained by multiple factors such as living environment, lifestyle, and health conditions (Reynolds et al., 2022). The rise of emotion theories and positive psychology has provided a theoretical framework for understanding the sources and functions of positive emotions in older adults (Koydemir et al., 2021). In the study of positive emotions, Physical activity has gradually become a focal variable. It not only contributes to improving the physical health of older adults but also has a positive impact on their psychological well-being and social relationships (San Román-Mata et al., 2020; An et al., 2020). It has been shown that physical exercise can significantly improve the sense of well-being and life satisfaction of older adults and alleviate their psychological problems to a certain extent by enhancing physical function, improving cognitive function, and promoting social interaction (Stevens et al., 2020; Hou et al., 2024). Meanwhile, physical activity has a natural social interaction attribute and is an important field for social capital construction (Jia et al., 2023). Whether individuals participate in physical activity, or watch sports competitions, physical activity can provide more opportunities for interpersonal interactions and group connections, enhance social participation, and promote social trust (Son et al., 2021; Wei et al., 2022). Therefore, social capital may act as a critical bridge linking physical activity among older adults to the improvement of positive emotions.

Based on this, the present study focuses on the positive emotions of older adults and explores the relationships among physical activity, social capital, and positive emotions. This study addresses two key questions. First, does physical activity enhance positive emotions in older adults? Second, can social capital play a mediating role between physical activity and positive emotions in older adults? By addressing these challenges, this research will not only advance theoretical perspectives on physical activity and social capital, but also yield practical evidence and policy recommendations for promoting mental health in older adults.

## 2 Literature review and research hypotheses

### 2.1 Physical activity and positive emotions in older adults

An increasing body of research evidence supports the link between physical activity and positive emotions (Kruk et al., 2019; Al Sudani, 2015). Physical activity refers to the intentional and planned physical activities, such as sports, leisure, and recreation, that individuals engage in to enrich their lives, promote physical and mental development, and strengthen social interactions (Borbón-Castro et al., 2020). In general, physical activity can be divided into two forms: direct participation, such as engaging in exercise, and indirect participation, such as watching sports events or purchasing sports equipment (Thurm et al., 2024). Participating in physical activity is one of the most convenient and enjoyable means of promoting physical and mental development. Long-term participation in Physical activity helps individuals maintain an appropriate body shape, prevent chronic diseases, thereby enhancing health levels and boosting resistance to illness (Izquierdo et al., 2021; Jin and Jing, 2024). Moreover, Physical activity brings psychological pleasure, as it is a voluntarily chosen form of “play,” “leisure,” or “game,” which increases positive emotions such as happiness, joy, and pleasure, while reducing negative emotions like anxiety, depression, and stress (Harikkala-Laihinen et al., 2022). Particularly, engaging in group physical activities helps reduce feelings of loneliness (Mansourian, 2021). Based on the above analysis, we propose Hypothesis 1:

**H1**: Physical activity has a significant positive effect on the positive emotions of older adults.

### 2.2 Social capital and positive emotions in older adults

Social capital is a complex concept in sociological research. Depending on the research perspective, studies on social capital can be categorized into three levels: micro, meso, and macro. At the micro level, the representative scholar Pierre Bourdieu defined social capital as “the aggregate of actual and potential resources owned by social members or groups” (Bourdieu, 2018). At the meso level, James Coleman described it as “the material that consists of certain aspects of social structures, which benefits specific actions of actors” (Coleman, 1988). At the macro level, Robert Putnam defined social capital as “certain characteristics of social organizations, such as trust, norms, and networks, which can enhance social efficiency by facilitating cooperative actions” (Putnam, 1993).

Previous scholars generally agree that social capital originates from social networks, and is a product of individuals' connections with the outside world. At the micro level, social capital focuses on the social network ties between individuals, thus micro-level social capital is regarded as individual social capital (Antheunis et al., 2015). Social capital at the meso and macro levels, however, emphasizes the connections between individuals and collectives or social organizations, and is thus considered as collective social capital (Claridge, 2020). Previous research on the measurement of individual social capital is relatively consistent, typically assessing it through inquiries about respondents' social network relationships. For example, Yan-Jie Bian used the “New Year's Visiting Network” to measure the social networks of urban residents in China, considering dimensions such as network size, network centrality, network heterogeneity, and network disparities. In terms of measuring collective social capital, previous studies have often relied on indicators such as trust, norms, and networks (Bian et al., 2024). For instance, Lin Nan measured social capital in American communities using multiple dimensions, including reciprocity, trust, ties (work ties, informal ties), and activities (volunteer work, charitable activities) (Lin and Dumin, 1986).

Numerous studies have shown that both individual-level and collective-level social capital are related to the emotions of older adults. Han found that social capital, including family support, trust, and reciprocity, has a significant association with the emotions of older adults (Han et al., 2018). Other scholars argue that both cognitive and structural social capital serve as protective factors for the positive emotions of urban older adults (Lu and Peng, 2019). English et al. also found that trust, reciprocity, and social networks are significantly associated with emotional changes in older adults, and that social capital can effectively suppress the increase in negative emotions among the elderly (English and Carstensen, 2014). The Health Production Theory posits that self-emotions are an investment good, influenced by factors such as genetic makeup, living environment, lifestyle, economic status, and social capital (Turner and Stets, 2006). Among these, social capital enables older adults to obtain social support through embedded social networks during their social participation, alleviating negative emotions such as loneliness, emptiness, and loss. It also provides emotional comfort, mental solace, and other forms of human care, enhancing their sense of gain, happiness, and pleasure. Therefore, the accumulation of social capital can improve mental health and directly reduce negative emotions. Based on this, we argue that social capital has an important impact on the positive emotions of older adults. Furthermore, we hypothesize that both individual social capital and collective social capital can influence the positive emotions of older adults. Based on the above analysis, we propose the following hypotheses:

**H 2:** Individual social capital has a significant positive effect on the positive emotions of older adults.
**H 2a:** Collective social capital has a significant positive effect on the positive emotions of older adults.

### 2.3 Physical activity, social capital, and positive emotions in older adults

Since the 1990s, Patnam has measured social capital in terms of the number of sports social groups and sports clubs and explored the role of participation in physical activity in fostering social capital, triggering a wide-ranging academic discussion on physical activity and social capital (Stern and Putnam, 1993). Numerous studies have shown that physical activity is an important way to broaden social networks and cultivate social capital. At the level of individual capital, individuals' participation in physical exercise helps to broaden the scope of interpersonal interactions and expand the size of social networks, thus enhancing individual social capital. For example, Woods found that when people with strong social ties exercise together, it generates “adhesion-type” social capital, and when people with weak social ties exercise together, it generates “bridging-type” social capital (Claridge, 2018). At the level of collective social capital, both the spontaneous formation of individual interest-oriented physical activity clubs and commercial, for-profit fitness venues provide the basis for fostering collective social capital (Kim et al., 2021). Putnam argues that “sports social group participation can promote social mutual trust through socialization, which in turn casts the level of trust of the residents and the society to go prosperous “(Putnam, 1997). Other scholars have found that urban residents choosing to participate in group exercise can promote the generation of collective social capital. In addition, achieving victory in a large sports group program relies on mutual cooperation and trust with participating members, and individuals who regularly participate in group programs rise to a much higher level of trust in others than those who do not participate in group programs (de Jeu and Stroet, 2021). Accordingly, we formulate Hypotheses 3 and 3a.

**Hypothesis 3:** Physical activity has a significant positive effect on individual social capital.
**Hypothesis 3a:** Physical activity has a significant positive effect on collective social capital.

**Figure 1:**
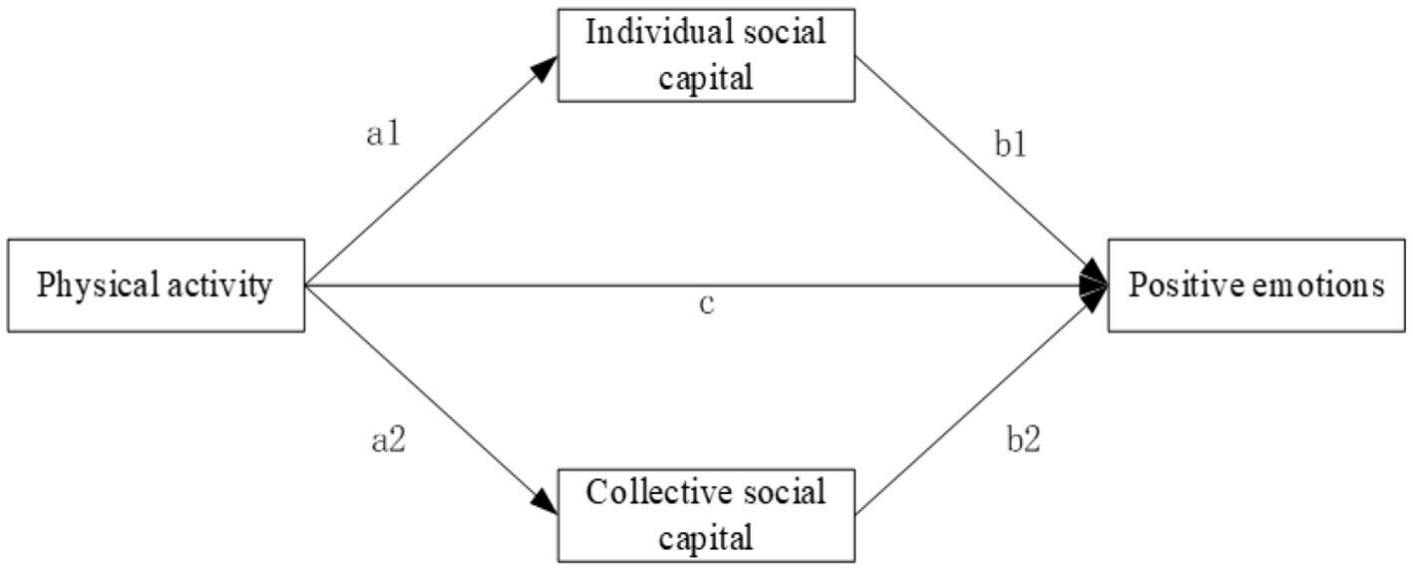
The proposed hypothetical model.

In the context of Chinese culture, different individuals form emotionally colored social bonds through their joint participation in Physical activity, which in turn constitute social capital. This social capital is characterized by strong connectivity, functional versatility, and frequent obligations. By fulfilling roles such as risk-sharing, emotional support, and identity recognition, it helps improve individuals' mental health, including the enhancement of positive emotions (Huang et al., 2021). First, at the individual social capital level, according to social capital theory, the larger an individual's social network, the broader the channels through which the individual can obtain information and access available social resources (Kong et al., 2022). Social networks can play an important role in helping individuals escape economic hardships, acquire job information, and increase personal income (Huang, 2021). Second, at the collective social capital level, reciprocity and social trust reflect the willingness of individuals to engage in social exchanges. In the Chinese context, influenced by Confucianism, interpersonal relationships emphasize the value of “love is worth a thousand in gold”. On the basis of mutual trust, individuals are highly motivated to offer favors to others, while also expecting reciprocal favors in return. This reinforces the emotional connections of reciprocity and mutual assistance. Trust relationships formed through long-term interactions, such as those based on kinship, can provide direct emotional support for both parties in the relationship (Marlier et al., 2015), and further promote the conversion of social capital into human capital. This helps individuals engage in more frequent interactions with other social members, thereby strengthening identity recognition, enhancing a sense of belonging, fostering a cooperative atmosphere, and promoting physical and mental health (Shiell et al., 2020). We believe that the Physical activity of older adults is influenced by social capital, and there is an intrinsic mechanism through which Physical activity enhances positive emotions in older adults, with social capital playing a mediating role. Based on this, we propose Hypothesis 4 and Hypothesis 4a. The research framework is shown in Figure 1.

**Hypothesis 4:** Individual social capital plays a mediating role in the relationship between Physical activity and positive emotions in older adults.
**Hypothesis 4a**: Collective social capital plays a mediating role in the relationship between Physical activity and positive emotions in older adults.

## 3 Materials and methods

### 3.1 Participants

The data for this study comes from the China Family Panel Studies (CFPS) database. The CFPS database covers a total of 27,001 people in 25 provinces in China in terms of economy, demographics, education, and health. Currently, the latest publicly available data year of the CFPS database is 2022, so this paper selects 2022 data as the research sample. Given that this study focuses on the elderly, only valid samples of 3,007 people over the age of 65 were retained, 1,441 of them were female and 1,556 were male, accounting for 47.92% and 52.08%, respectively.

### 3.2 Measures

**Dependent variable:** This study focuses on the emotional effects of Physical activity on older adults, taking into account the emotional impact of individual participation in Physical activity in real life. Following the approach used in previous studies (Fingerman et al., 2021; Xu et al., 2019), we constructed the positive emotion indicator for older adults based on two dimensions: “I feel pleasant” and “I live a very happy life.” This individual positive emotion indicator system characterizes the emotional impact of Physical activity on older adults, providing a measurement tool for the development of targeted policies. Specifically, the CFPS survey uses two self-assessment items: “I feel pleasant” and “I live a very happy life.” The response scale for “I feel pleasant” is defined as follows: 1 = almost never, 2 = sometimes, 3 = often, 4 = very often. The response scale for “I live a very happy life” is defined as: 1 = almost never, 2 = sometimes, 3 = often, 4 = very often.

Based on the determination of the sub-dimension indicators, the steps for constructing the positive emotion indicator for older adults are as follows:

**Step 1**: Normalize the sub-dimension indicators so that the mean of each indicator falls within the range [0, 1]. The specific formula is as follows:
  (1) $$\begin{array}{l} x_{\rho }=\frac{\chi -\chi_{\min}}{\chi_{\max}-\chi_{\min}} \end{array}$$
  Where χ represents the classification into dimensional indicators and happy and pleasant, χ_max_ represents the maximum value and χ_min_ represents the minimum value of the indicator.
**Step 2**: Confirm the weights. Enhancing positive emotions in older adults should focus on the collaborative development of various aspects, and equal weight should be assigned to each dimension. Therefore, the equal weight method is used to assign weights to the two dimensions of “feeling happy” and “feeling pleasant,” with the corresponding average weight for each dimension being 1/4.
**Step 3**: Construct the positive emotion indicator for older adults using the average Euclidean distance method. Compared to principal component analysis, factor analysis, and coefficient of variation methods, the average Euclidean distance method calculates the distance between each sample's actual value and both the optimal and worst values. This makes the indicator system more intuitive and offers advantages that cannot be replaced by linear summation methods (Sethy, 2016). The calculation formula for the average Euclidean distance method is as follows:
  (2) $$\begin{array}{l} \text{Positive emotion}{\text{s}}_{1}=\frac{\sqrt{\text{Happy feelin}{\text{g}}^{2}+\text{Pleasur}{\text{e}}^{2}}}{\sqrt{4}} \end{array}$$
  (3) $$\text{Positive }{\text{emotions}}_{\text{2}}\text{=}\frac{\sqrt{{\left(\text{1-Happy feelin}\right)}^{\text{2}}\text{+}{\left(\text{1-Pleasure}\right)}^{\text{2}}}}{\sqrt{\text{4}}}$$
  (4) $$\text{Positive emotions = }\frac{\text{Positive }{\text{emotions}}_{\text{1}}\text{+ Positive }{\text{emotions}}_{\text{2}}}{\text{2}}$$

It is important to note that after standardizing the dependent variable, it is entirely normal for the maximum and minimum values to be non-integer, as during the standardization process, the data is transformed into a continuous variable with a mean of 0 and a standard deviation of 1. The purpose of standardizing is to ensure that the variable meets the requirements of the linear analysis model, and non-integer values do not affect the analysis results.

**Independent Variable:** Physical activity. A binary dummy variable for Physical activity is constructed based on the number of times the respondent exercised in the past week. Specifically, “0 = did not engage in Physical activity, 1 = engaged in Physical activity”.

**Mediator Variable**: Social Capital. Specifically, it includes two dimensions: individual social capital and collective social capital. Drawing on previous CFPS research, both individual social capital and collective social capital were measured (Huang et al., 2019). In the CFPS survey, respondents were asked the question “How good are your interpersonal relationships?” and were scored on a scale from low to high, with a range of 0–10. The higher the score, the stronger the individual's social capital. Collective capital includes two items: reciprocity and trust. Based on the question “Are most people helpful or selfish?” a binary dummy variable for reciprocity was constructed. Those who answered “Most people are helpful” were coded as “1 = reciprocal,” and those who answered “Most people are selfish” were coded as “0 = non-reciprocal.” Based on the question “Do you trust others or are you skeptical?” a binary dummy variable for trust was constructed. Those who answered “Most people can be trusted” were coded as “1 = trustworthy,” and those who answered “It's better to be cautious” were coded as “0 = non-trustworthy.” The Cronbach's alpha coefficient for collective social capital is 0.83, which has a high reliability. Finally, both individual social capital and collective social capital were standardized.

**Control Variables**: These include gender “0 = female, 1 = male”, age as a continuous variable, household registration (“0 = rural household, 1 = urban household”), marital status “0 = single status (unmarried, divorced, or widowed), 1 = non-single status (married or cohabiting)”, health status “1 = poor, 2 = average, 3 = very healthy”, education level “0 = below primary school, 1 = primary school, 2 = middle school, 3 = high school, 4 = college or higher”, and income level “1 = low income, 2 = middle income, 3 = high income”.

### 3.3 Statistical analysis

First, the Euclidean distance method was used to construct the positive emotion indicator for older adults, and descriptive statistics and correlation analysis were conducted for the relevant variables involved in this study. Next, Structural Equation Modeling (SEM) was employed to establish a parallel mediation model to analyze the mediating effect of social capital in the relationship between Physical activity and positive emotions in older adults. The Bootstrap method (1,000 repetitions) was used for resampling the sample data to estimate the direct and indirect effects of the core variables and construct the confidence intervals for these effects. When the confidence interval does not include 0, the effect is considered significant. The model fit indices include the ratio of chi-square statistic to degrees of freedom (χ^2^/DOF, < 3), Comparative Fit Index (CFI, >0.90), Tucker-Lewis Index (TLI, >0.90), Root Mean Square Error of Approximation (RMSEA, < 0.08), and Standardized Root Mean Square Residual (SRMR, < 0.10). Meanwhile, in order to test the potential effect of gender on mediated paths, this study used Multi-group Structural Equation Modeling (Multi-group SEM) to divide the samples into two groups of males and females, and to compare the between-group differences in path coefficients by Likelihood Ratio Test (LRT). Finally, the average treatment effect of ATT, i.e., the experimental group, was obtained through the propensity score matching test. All statistical methods were performed using STATA 17.0 software.

## 4 Results

### 4.1 Descriptive statistical analysis

Table 1 presents the descriptive statistics of all variables. The results show that the mean value of elderly people's positive emotions is 0, with a standard deviation of 1. The minimum value is −1.137 and the maximum value is 2.296. The negative skewness of this indicator suggests that most elderly people have a high level of positive emotions. Among the total sample, 59.9% of elderly individuals engage in regular Physical activity. Additionally, 58.9% have a high level of individual social capital, and 47.9% have a high level of collective social capital. In terms of demographics, 52.1% of the sample are male, while 47.9% are female. The average age is 71.78 years, with ages ranging from 66 to 97. A greater proportion of elderly individuals live in urban areas (67.8%) compared to rural areas (32.2%). Regarding marital status, 81.7% of the elderly are married, while 18.3% are single, divorced, or widowed. The elderly individuals' physical health status is generally good, though their educational level is relatively low. The mean income level is 1.785, with a standard deviation of 0.803, indicating that most elderly people fall into the middle- to low-income group.

**Table 1:** Basic variable description statistics table.

**Variable**	***N***	**Mean**	**Std. Dev**.	**Min**	**Max**
Positive emotions	3,007	0	1	−1.137	2.296
Physical activity	3,007	0.599	0.490	0	1
Individual social capital	3,007	0.589	0.492	0	1
Collective social capital	3,007	0.479	0.5	0	1
Gender	3,007	0.521	0.5	0	1
Age	3,007	71.783	4.887	66	97
Urban	3,007	0.322	0.467	0	1
Marital	3,007	0.817	0.386	0	1
Health	3,007	2.08	0.683	1	3
Edu	3,007	1.49	0.802	1	4
Income	3,007	1.785	0.803	1	3

### 4.2 Correlation analysis

Table 2 presents the Pearson correlation analysis results between Physical activity, social capital, and positive emotions in elderly individuals. The analysis reveals that, excluding control variables, there are significant positive correlations between physical activity, individual social capital, collective social capital, and positive emotions, with statistically significant correlation coefficients. Specifically, physical activity is positively correlated with positive emotions in elderly individuals. Individual social capital is positively correlated with positive emotions but does not show a significant correlation with physical activity. Collective social capital is significantly positively correlated with positive emotions and physical activity. Overall, these results suggest that both physical activity and social capital have a certain impact on positive emotions in elderly individuals, but physical activity has a more significant effect on collective social capital.

**Table 2:** Correlation analysis results.

**Variables**	**Positive emotions**	**Physical activity**	**Individual social capital**	**Collective social capital**
Positive emotions	1.000			
Physical activity	0.131^***^	1.000		
Individual social capital	0.100^***^	0.025	1.000	
Collective social capital	0.064^***^	0.045^***^	0.092^***^	1.000

^***^p < 0.001.

### 4.3 Structural equation modeling (SEM) modeling

**Figure 2:**
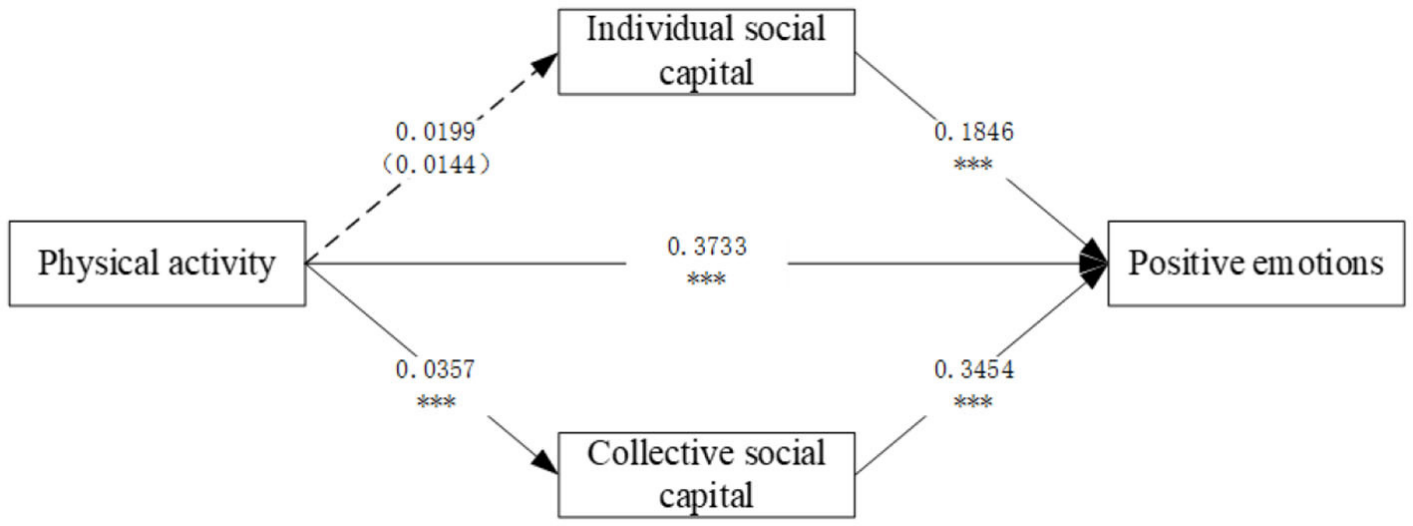
The impact of physical activity on individual social capital, collective social capital, and older adults' positive emotions. All estimates are standardized. **Solid lines** indicate significant effects, while **dashed lines** indicate non-significant effects. ***p < 0.001.

The SEM results show a reasonable and good fit with the data: χ^2^/DOF = 796.185/504 = 1.579, CFI = 0.976, TLI = 0.971, RMSEA = 0.089 (90% C.I. = 0.061–0.121), SRMR = 0.029. Table 3 shows the results of the standardized coefficients for the overall, direct and indirect effects of physical activity on positive emotions in older adults through social capital; Figure 2 shows the coefficients of the effects of each direct path.

**Table 3:** Path-coefficients of the mediating models.

**Paths**	**UC(SE)**	**95%CI**	**β**	**Cohen's *d***
**Total effect**				
PE → Pa	0.387 (0.001)	[0.283,0.490]	0.451 (0.006)	0.255
**Direct effects**				
PE → Pa	0.373 (0.001)	[0.269,0.477]	0.435(0.004)	0.235
**Indirect effects (total)**	0.014 (0.006)	[0.004,0.017]	0.016(0.003)	0.182
PE → ISC → Pa	0.007 (0.166)	[−0.001,0.015]	0.008(0.173)	–
PE → CSC → Pa	0.007 (0.005)	[0.001, 0.013]	0.008(0.009)	0.175

Effect sizes were calculated as the ratio of the total mediated effect, β = fully standardized coefficient; UC, unstandardized coefficient; SC, standardized coefficient; SE, standard error; PE, physical exercise; Pa, Positive affect; ISC, individual social capital; CSC, collective social capital.

As shown in Table 3, the total effects analysis indicated that the total effect of physical activity on positive mood was significant and large (*B* = 0.387, SE = 0.001, β = 0.451,95% CI[0.283,0.490], Cohen's *d* = 0.255), suggesting that for every one standard deviation increase in the level of physical activity, the positive mood level correspondingly increased by 0.387 standard deviations.

As shown in Figure 2, both individual social capital and collective social capital have a significant positive impact on older adults' positive emotions (β = 0.1846, *P* < 0.001; β = 0.3453, *P* < 0.001). This suggests that the enhancement of social capital, whether at the individual or collective level, contributes to improving older adults' positive emotions, with the influence of collective social capital being more significant. Therefore, Hypothesis 2 and Hypothesis 2a are confirmed.

Figure 2 also shows that physical activity does not have a significant effect on individual social capital (β = 0.0199, *P* = 0.166), while it has a significant positive impact on collective social capital (β = 0.0357, *P* = 0.014). In other words, physical activity is more effective in promoting social connections and interactions at the collective level for older adults, rather than simply increasing social resources at the individual level. This result highlights the social nature of physical activity, suggesting that participation in physical activities is more likely to strengthen the bond between older adults and the collective through interaction and cooperation at the collective level. Therefore, Hypothesis 3 is not confirmed, while Hypothesis 3a is confirmed.

The total indirect effects and all specific indirect utilities of the two mediating variables are also presented in Table 3. In terms of indirect effects, collective social capital had a significant positive effect on positive emotions among older adults (*B* = 0.007, SE = 0.005, β = 0.008, 95% CI [0.001, 0.013], Cohen's *d* = 0.175), suggesting that collective social capital partially mediates the relationship between physical activity and positive emotions, possibly through enhanced group belonging or resource sharing. In contrast, the mediating effect of individual social capital did not reach statistical significance (*B* = 0.007, SE = 0.166, β = 0.008, 95% CI [−0.001, 0.015]), and the confidence intervals contained zero values, suggesting that the mediating role of individual social capital was unstable or interfered with by sample heterogeneity. These findings validate Hypothesis 4a but do not support Hypothesis 4. Overall, only one of the indirect paths through social capital is significant: i.e., physical activity → collective social capital → positive emotions in older adults, and therefore, we can conclude that collective social capital mediates the relationship between physical activity and positive emotions in older adults.

### 4.4 Heterogeneity analysis by gender

Given that social capital and positive emotions may vary by gender, they were analyzed by subgroups (males vs. females) to avoid gender-related bias (see Table 4). The subgroup results indicated significant gender differences in the pathways through which physical activity influences positive emotions through collective social capital (β = 0.009, *P* = 0.032 between groups): in the male group, the mediating effect of collective social capital was stronger (β = 0.012,95% CI [0.005, 0.019]), possibly reflecting the fact that males are more reliant on group-based physical activity to build social bonds, while the Individual social capital path coefficient was higher in the female group (β = 0.011 vs. 0.005), which, although did not reach a significant difference (*P* = 0.087), suggests that women's positive mood may be influenced by individual exercise.

**Table 4:** Comparison of standardized path coefficients by gender grouping.

**Trails**	**Male β (SE)**	**Female β (SE)**	**Between-group differences β**	***P***
**Direct effects**				
PE → Pa	0.428 (0.013)	0.442 (0.015)	−0.014	0.012
**Intermediary pathway impacts**				
PE → ISC → Pa	0.005 (0.002)	0.011 (0.003)	−0.006	0.087
PE → CSC → Pa	0.012 (0.004)	0.003 (0.002)	0.009	0.007

PE, physical exercise; Pa, Positive affect; ISC, individual social capital; CSC, collective social capital.

### 4.5 Propensity score matching test

In order to address the issue of further self-selective bias in physical activity among older adults, Propensity Score Matching (PSM) was performed by balancing the treatment group (physical activity participants) and the control group (non-participants). The results showed (Table 5) that participation in physical activity had a significant positive effect on individuals' positive emotions. In the unmatched sample, the positive mood scores of the treatment group (participants) were significantly higher than those of the control group (non-participants), with a mean treatment effect (ATT) of 0.346 (SE = 0.037, *t* = 9.44, *p* < 0.001). After 1:15 nearest neighbor matching was used to control for selection bias, the ATT decreased to 0.274 (SE = 0.058, *t* = 4.72, *p* < 0.001), but the treatment effect remained highly statistically significant. This further illustrates the robustness of the results of this study.

**Table 5:** Propensity score matched treatment effect estimates (ATT).

**Sample type**	**Treated mean**	**Control mean**	**ATT**	**SE**	***t*-value**
Unmatched sample	0.140^***^	−0.208	0.346	0.037	9.44
Matched sample	0.139^***^	−0.135	0.274	0.058	4.72

^***^p < 0.001; ATT, Average treatment effect on the treated.

## 5 Discussion

In the context of global aging, this study examines the impact of physical activity on older adults' positive emotions based on data from the China Family Panel Studies (CFPS). Although previous research has already established a relationship between physical activity and positive emotions in older adults, the underlying mechanisms of this process remain to be explored. Therefore, we proposed a parallel mediation model to test the role of social capital. Several important conclusions can be drawn from this study.

First, we found that physical activity has a significant positive impact on older adults' positive emotions, a conclusion that aligns with existing literature. Starting from self-determination theory, academics have argued that physical activity can satisfy human autonomy, sense of being alive, and sense of belonging, explaining the relationship between an individual's motivation and emotional experience during physical activity (Kirkland et al., 2011). Numerous studies have also shown that physical activity can influence positive emotions in multiple dimensions by improving physical health, enhancing self-efficacy, and boosting psychological resilience (Gubert and Hannan, 2021; Stillman et al., 2020). On the other hand, physical activity stimulates the release of neurotransmitters such as dopamine and endorphins, and these physiological changes are believed to be an important mechanism for enhancing mood states (Zheng, 2022). In addition, emotion regulation theory suggests that physical activity is a means of emotion regulation by altering the level of physiological arousal, thereby regulating emotions, and when people are in a negative emotional state, participation in physical activity can increase physiological arousal and, in combination with situational factors and individual cognitive interpretations, transform it into a positive emotion (Huang et al., 2020). Compared with previous studies on the general population, the present study focused on the elderly population, further validating the generalizability and importance of physical activity on positive emotions in older adults. Older adults are more likely to face loneliness and low mood due to physical decline and social role changes, and physical activity provides a way for them to improve their mood (Bar-Tur, 2021). Particularly among older adults, participation in moderate physical activity not only enhances physical fitness, but also provides a sense of accomplishment and meaning in life, thereby enhancing overall well-being. Notably, the results of this study support the key role of exercise frequency in mood improvement, suggesting the value of regular physical activity among older adults. This finding provides an empirical basis for health interventions, suggesting that when promoting physical activity in older populations, there is a need to focus not only on improvements in physical health, but also to emphasize its positive effects on mental health and mood regulation. Future studies can further explore the differential effects of different types and intensities of exercise on the emotional state of older adults, providing a scientific basis for the development of more precise exercise programs.

Secondly, this study found that both dimensions of social capital (i.e., individual social capital and collective social capital) have a significant positive impact on older adults' positive emotions. Specifically, both individual-level social capital and collective-level social capital can enhance older adults' positive emotions. On the one hand, individual social capital provides emotional and instrumental support through interpersonal relationships, helping older adults obtain psychological comfort when facing loneliness or life stress. For example, Helliwell and Barrington-Leigh (2012) pointed out that individuals with strong social connections are more likely to perceive higher life satisfaction (Helliwell and Barrington-Leigh, 2012). On the other hand, collective social capital promotes positive social interactions and mental health by enhancing older adults' sense of belonging and identification with their community or group. This collective interaction helps eliminate potential feelings of alienation and enhances their sense of value and belonging (Lambert et al., 2013). It is worth noting that the results of this study show that collective social capital has a more significant effect on older adults' positive emotions compared to individual social capital. This may be related to the intrinsic characteristics of physical activity and collective activities. Engaging in physical activity not only enhances physical health but also provides older adults with a platform for interaction and cooperation, promoting mutual support and trust through shared goals (Milligan et al., 2004). This finding also validates the core role of social capital in emotional regulation, as highlighted in previous studies, which suggests that social interactions and support systems strengthen emotional regulation capacity (Lakey and Orehek, 2011). Overall, this study reveals the importance of social capital in enhancing older adults' positive emotions, providing practical guidance for improving their emotional health. The findings suggest that future public health interventions should focus on promoting older adults' social participation and community integration to enhance their social capital, thereby improving their psychological well-being.

Finally, this study examined the mediating effects of social capital (i.e., individual social capital and collective social capital) in the relationship between physical activity and older adults' positive emotions. The results show that although both dimensions of social capital have a significant positive impact on older adults' positive emotions, we found that physical activity has a significant effect only on collective social capital, but not on individual social capital. Moreover, the mediation analysis revealed that only collective social capital plays a significant mediating role in this relationship. This finding highlights the collective nature of physical activity, as it enhances older adults' sense of community belonging and social support through participation in group activities and social interactions, thereby improving their positive emotional well-being. This aligns with the “bridging” theory of social capital, which posits that collective social capital primarily helps individuals obtain more external support resources by expanding social networks and fostering social connections (Frey and Eitzen, 1991). For example, older adults who engage in group activities such as square dancing or Tai Chi, through interaction with others, can experience emotional recognition and social support, which to some extent alleviates feelings of loneliness and negative emotions (Ouyang et al., 2024; Zhou et al., 2024). In contrast, the impact of physical activity on individual social capital is not significant, which may reflect the characteristics of older adults' life situations. Individual social capital often relies on deep emotional support and long-term relationships, which are more likely to come from family members or close relationships, rather than short-term social contacts formed through physical activities (Zimmer et al., 2023). This result may be due to the unique role of collective social capital in facilitating resource sharing and social cohesion, whereas individual social capital focuses more on aspects such as resource acquisition and utilization at the individual level. For example, it has been shown that certain characteristics of collective social capital in terms of structural and cognitive dimensions (e.g., mutual trust among members, shared values, etc.) allow it to play a mediating role in moderating the relationship between physical activity and positive emotions (Liang et al., 2020), whereas individual social capital is unable to achieve such a mediating effect due to its relative independence and decentralized nature (Chen et al., 2019).

Furthermore, the multi-group analysis in this study revealed gender heterogeneity in the mediating role of social capital between physical activity and positive affect. Significant negative effects were observed between male and female groups, with the mediating effect of collective social capital being significantly stronger in the male population. While individual social capital showed non-significant mediating effects in both genders, the path coefficient was higher among females. These findings align with the expectations of social role theory (Storr et al., 2022): men typically rely more on group-based activities (e.g., team sports, community organizations) to establish social connections, whereas women may prioritize dyadic interactions (e.g., exercising with friends) to obtain emotional support (Feil et al., 2022). Older female adults may be more inclined to perceive physical exercise as a means to improve physical health rather than a tool to foster group relationships, which also limits its role in enhancing collective social capital. In summary, these findings deepen our understanding of the social function of physical exercise and provide practical implications for improving positive affect in older adults. On one hand, interventions should focus on helping older adults build collective social capital to enhance mental health. Meanwhile, given the limited role of individual social capital, family-involved physical activities could be promoted to strengthen interactions within close-knit networks, compensating for the shortcomings of exercise in this dimension. On the other hand, as globalization accelerates, older adults' positive affect is influenced by multiple factors. Understanding the unique interplay between collective social capital and physical exercise among Chinese older adults not only aids in designing culturally tailored policies and interventions but also contributes a distinct Chinese perspective to global aging and health research.

Despite using the latest China Family Panel Studies (CFPS) data, there are still some limitations in our study. First, this study used cross-sectional data and estimated the relationships between variables using structural equation modeling (SEM), but there are limitations that do not allow for a clear inference of causality. Looking more deeply, the data collection process may have been somewhat biased. For example, there may be an association between individuals' levels of positive affect and physical activity and social engagement, whereby those with higher positive affect are more inclined to engage in physical activity and report higher levels of social engagement. This association may affect the accuracy of the data and generalizability of the findings. Therefore, we consider further validating the causality of these relationships in future studies through longitudinal designs or experimental methods. Future research could employ longitudinal designs or experimental methods to further validate the causality of these relationships. Second, our study primarily focuses on the two dimensions of social capital—individual and collective social capital—without fully covering other aspects of social capital, such as structural social capital or cognitive social capital. Third, the study only used “frequency of exercise” to measure physical activity, and future research could use more detailed indicators such as “hours of physical activity,” “mode of physical activity,” and “watching sports games” by distributing questionnaires. Future research could analyze this by distributing questionnaires using more detailed indicators such as “hours of physical activity,” “types of physical activity,” and “watching sports games.

## 6 Conclusion

Based on data from the China Family Tracking Survey (CFPS) database, this study incorporates physical activity, social capital, and positive emotions of older adults within the same framework and focuses on the mediating effects of different dimensions of social capital between physical activity and positive emotions of older adults. The study found that (1) physical activity has a significant positive effect on positive emotions of older adults; (2) two dimensions of social capital (individual social capital and collective social capital) have a significant positive effect on positive emotions of older adults; (3) physical activity only has a significant positive effect on collective social capital; and the mediating effect shows that only collective social capital plays a significant mediating effect between physical activity and positive emotions of older adults. In summary, given the significant impact of collective social capital on positive emotions among older persons, on the one hand, the Chinese Government should further promote the social ties and collective interactions of older persons with the help of physical exercise through the provision of public venues, the organization of community activities and the strengthening of social interaction platforms; on the other hand, the link between physical activity, collective social capital and positive emotions should be publicized to the elderly, so that they can understand that the formation of collective social capital through participation in physical activity can enhance their positive emotions. Increasing physical activity among older persons through these initiatives will allow the value of physical activity among older persons to be more deeply realized and will help to achieve the goal of active aging.

## Data Availability

The original contributions presented in the study are included in the article/supplementary material, further inquiries can be directed to the corresponding author.
